# Colistin-Conjugated Selenium Nanoparticles: A Dual-Action Strategy Against Drug-Resistant Infections and Cancer

**DOI:** 10.3390/pharmaceutics17050556

**Published:** 2025-04-24

**Authors:** Mais E. Ahmed, Kholoud K. Alzahrani, Nedal M. Fahmy, Hayfa Habes Almutairi, Zainab H. Almansour, Mir Waqas Alam

**Affiliations:** 1 Department of Biology, College of Science, University of Baghdad, Jadriya, Baghdad 10071, Iraq; 2 Department of Biology, University College of Umluj, University of Tabuk, Umluj 71491, Saudi Arabia; 3 Department of Chemistry, College of Science, King Faisal University, P.O. Box 380, Al Ahsa 31982, Saudi Arabia; halmutairi@kfu.edu.sa; 4 Department of Biological Sciences, College of Science, King Faisal University, Al Ahsa 31982, Saudi Arabia; 5 Department of Physics, College of Science, King Faisal University, Al Ahsa 31982, Saudi Arabia

**Keywords:** selenium nanoparticles, colistin, *Pseudomonas aeruginosa*, antimicrobial resistance, *Candida* spp., MCF-7breast cancer

## Abstract

**Background/Objective**: Antimicrobial resistance (AMR) and therapy-resistant cancer cells represent major clinical challenges, necessitating the development of novel therapeutic strategies. This study explores the use of selenium nanoparticles (SeNPs) and colistin-conjugated selenium nanoparticles (Col-SeNPs) as a dual-function nanotherapeutic against multidrug-resistant *Pseudomonas aeruginosa*, antifungal-drug-resistant *Candida* spp., and human breast carcinoma (MCF-7) cells. **Methods**: SeNPs were synthesized and characterized using UV-Vis spectroscopy, atomic force microscopy (AFM), energy-dispersive X-ray spectroscopy (EDX), X-ray diffraction (XRD), field emission scanning electron microscopy (FESEM), transmission electron microscopy (TEM), and Fourier-transform infrared spectroscopy (FTIR), confirming their nanoscale morphology, purity, and stability. **Results**: The antimicrobial activity of SeNPs and Col-SeNPs was assessed based on the minimum inhibitory concentration (MIC) and bacterial viability assays. Col-SeNPs exhibited enhanced antibacterial effects against *P. aeruginosa*, along with significant downregulation of the *mexY* efflux pump gene, which is associated with colistin resistance. Additionally, Col-SeNPs demonstrated superior antifungal activity against *Candida albicans*, *C. glabrata*, and *C. krusei* compared to SeNPs alone. The anticancer potential of Col-SeNPs was evaluated in MCF-7 cells using the MTT assay, revealing dose-dependent cytotoxicity through apoptosis and oxidative stress pathways. Although MCF-7 is not inherently drug-resistant, this model was used to explore the potential of Col-SeNPs in overcoming resistance mechanisms commonly encountered in cancer therapy. **Conclusions**: these findings support the promise of Col-SeNPs as a novel approach for addressing both antimicrobial resistance and cancer treatment challenges. Further in vivo studies, including pharmacokinetics and combination therapies, are warranted to advance clinical translation.

## 1. Introduction

According to Ferrara et al. (2024), the growth of antimicrobial resistance (AMR) poses a substantial risk to world health [1]. The rise of multidrug-resistant infections has severely limited therapeutic options [2]. Notably, *Pseudomonas aeruginosa* has been identified as a high-priority opportunistic pathogen, frequently related to serious nosocomial infections, such as ventilator-associated pneumonia, bloodstream infections, and chronic wound infections [3]. The WHO assigned *P. aeruginosa* as a critical priority pathogen based on its intrinsic resistance mechanisms and rapid development of its resistant phenotypes to the last-line antibiotics, including carbapenems and colistin [4,5]. The overexpression of efflux pumps, especially the MexXY–OprM system, plays a very important role in antibiotic failure, which urges the identification of novel therapeutic strategies [6]. New strategies are required that can transcend these traditional avenues of resistance, with a specific emphasis on nanotechnology-based therapy [7,8].

Recent advances in the use of nanoparticles have opened new avenues for therapeutic interventions targeting microbial resistance [9]. Their unique physicochemical characteristics allow the development of novel strategies to overcome microbial defense mechanisms [10,11]. Among these, selenium nanoparticles (SeNPs) have emerged as particularly promising due to their combination of biological efficacy, excellent biocompatibility, and low toxicity [12,13]. Selenium, an essential trace element, plays critical roles in antioxidant defense, immune modulation, and cellular homeostasis. When engineered at the nanoscale, selenium exhibits enhanced pharmacokinetic and pharmacodynamic properties that significantly boost its biomedical potential.

SeNPs demonstrate pleiotropic pharmacological activities—including antimicrobial, antifungal, and anticancer effects—which are advantageous for addressing multidrug resistance. Studies have shown that SeNPs disrupt bacterial membranes, interfere with metabolic functions, and generate reactive oxygen species (ROS), leading to broad-spectrum antimicrobial effects [14,15]. Additionally, SeNPs modulate microbial signaling and inhibit efflux pumps, a key mechanism of antibiotic resistance. Their selective cytotoxicity toward cancer cells, with minimal harm to normal tissues, further highlights their potential as a safer alternative to conventional chemotherapies. These multifunctional properties justify the selection of SeNPs in this study as the foundation for a dual-action nanotherapeutic platform aimed at drug-resistant infections and cancer.

They have great penetration in biofilms and the tendency to inhibit the efflux pumps, thus being mainly attractive in approaches against MDR bacterial infections [14,16,17]. Nanoparticles, e.g., silver nanoparticles (Ag NPs), become highly effective, mostly when proposed in synergistic combination, conjugated, with known antibiotics, such as colistin, an antibiotic of last resort used against MDR *P. aeruginosa* [18]. Thus, Col-SeNPs, the conjugation of colistin with SeNPs, putatively represent a dual-action strategy whereby membrane disruption mediated by colistin is combined with intracellular and metabolic disruption mediated by SeNPs, thus overcoming resistance mechanisms mediated by MexXY [19,20].

The rationale for using colistin with metal nanoparticles is not just their individual antimicrobial effects but also their capacity to act synergistically [18,21]. Colistin’s cationic characteristics allow for excellent interactions with bacterial membranes, increasing the penetration and cellular uptake of nanoparticles, like silver nanoparticles (Ag NPs) and selenium nanoparticles (SeNPs) [22]. Recent research suggests that functionalizing nanoparticles with antibiotics can alter the stress response in bacteria, reducing the likelihood of antibiotic resistance emergence [23]. As a result, Col-SeNPs may provide a multimodal strategy to bacterial infection control, utilizing synergistic processes, such as membrane destabilization, metabolic inhibition, and efflux pump inhibition. In addition to their potential antimicrobial role, SeNPs have also been explored for their anticancer properties, especially against the human breast cancer cells MCF-7 [24]. SeNPs exhibited selective cytotoxicity toward tumor cells by using different mechanisms: induction of apoptosis, mitochondrial dysfunction, and modulation of oxidative stress [25,26]. The combination of SeNPs with colistin therefore might result in enhanced therapeutic efficacy due to higher cellular uptake, changes in membrane permeability, or the induction of synergistic cytotoxic pathways [15,27]. The evaluation of Col-SeNPs is of immense biomedical relevance in view of the global burden in the management of breast cancer, especially due to chemoresistance and toxicities associated with conventional therapies [15].

In addition to bacterial infection and cancer treatment, SeNPs have been reported to exhibit antifungal properties, especially against clinically relevant *Candida* species [28,29,30]. An increased incidence of azole-resistant *Candida* strains has raised a need for the consideration of alternative antifungal strategies [31]. In this respect, chitosan-functionalized SeNPs showed higher antifungal efficacy than SeNPs alone by inducing a dose-dependent increase in growth inhibition for all the *Candida* spp. tested [32]. On the other hand, the combination of colistin with nanoparticles, e.g., Col-SeNPs, potentially represents a highly promising candidate with great potential to function through cell wall disruption, the induction of oxidative stress, and changes in membrane permeability for antifungal applications [33,34].

The study aims to reduce *mexY* expression in *P. aeruginosa*, boosting the bacteria’s susceptibility to colistin while also improving its antifungal and anticancer efficiency. This novel dual-function system combines nanotechnology with antibacterial and anticancer tactics, offering a potentially viable therapeutic option. The current study aims to synthesize and characterize selenium nanoparticles (SeNPs), with a focus on their antibacterial, antifungal, and anticancer effects. *P. aeruginosa* isolates will be identified first from clinical samples; then, SeNPs will be synthesized and functionalized with colistin to produce colistin-conjugated SeNPs (Col-SeNPs). Therefore, the in vitro antimicrobial efficacy of Col-SeNPs will be determined via MIC determinations, bacterial viability tests, and the study of modulating *mexY* gene expression in colistin-resistant *P. aeruginosa*. The antifungal activity will be performed against *Candida* spp., while the anticancer potentiality will be assessed through MTT assays by utilizing MCF-7 breast cancer cells. We also carried out experiments to observe if Col-SeNPs increase cytotoxicity by inducing apoptosis and oxidative stress.

The laboratory study has possible implications for clinical settings, particularly in the treatment of multidrug-resistant *P. aeruginosa* and fungal infections. The proposed use of colistin, a last-resort antibiotic, in combination with nanoparticle conjugation may reduce its toxicity while increasing its therapeutic efficacy. Furthermore, ongoing advances in nanomedicine may facilitate tailored delivery mechanisms, reducing off-target effects and improving the therapeutic index. As a result, this study coincides with the overall goal of introducing nanotechnology into antibacterial and oncological therapy frameworks. This study examined Col-SeNPs in relation to bacterial resistance, fungal infections, and cytotoxic effects on cancer cells. We present a new dual-function strategy that combines nanotechnology and standard antimicrobial treatment, with the potential to improve future therapeutic breakthroughs in infectious disease management, antifungal interventions, and oncology. Most importantly, our observations will further elucidate the role of Col-SeNPs as a next-generation therapeutic agent in building the foundation for further mechanistic and translational research on AMR, fungal pathogenesis, and cancer therapeutics.

## 2. Materials and Methods

### 2.1. Isolation and Identification of Pseudomonas aeruginosa

#### 2.1.1. Sample Collection and Bacterial Isolation

Ten clinical samples of *Pseudomonas aeruginosa* were collected from patients in hospitals in Baghdad, Iraq, who had wound and burn infections. The isolates were cultured on MacConkey agar and Cetrimide agar (selective and differential media) to confirm their ability to grow under conditions favorable for *P. aeruginosa*. The colonies were examined for their morphological characteristics, including a mucoid appearance, pigmentation, and fluorescence under UV light, which are typical features of *Pseudomonas* species. Additionally, pathogenic fungal isolates, including *Candida albicans*, *Candida guilliermondii*, and *Candida ciferrii,* isolated from urine, were generously provided by the College of Medicine at Iraqi University in Baghdad, Iraq, with all isolation identifications performed using the VITEK^®^ 2 System [35]. All microbiological work was conducted in accordance with biosafety level 2 (BSL-2) guidelines. Standard safety protocols were followed, including the use of personal protective equipment (PPE), proper sterilization of work surfaces and instruments, and safe disposal of biohazardous materials.

#### 2.1.2. Biochemical Identification and Antibiotic Susceptibility Testing

The isolates were further confirmed using the VITEK^®^ 2 Compact automated system (bioMérieux), which utilizes biochemical profiling for species identification [36]. The antibiotic susceptibility profile of each isolate was also determined using the VITEK^®^ 2 AST-GN card, which measures the minimum inhibitory concentration (MIC) of multiple antibiotics, including colistin.

The clinical *Candida* isolates used in this study—*Candida albicans* (R3), *Candida guilliermondii* (R4), and *Candida ciferrii* (R7)—were kindly provided by the College of Medicine, Iraqi University (Baghdad, Iraq). Species-level identification was conducted using the VITEK^®^ 2 Compact system (bioMérieux), with high confidence in classification. According to the donor institution’s diagnostic records, all three isolates exhibited resistance to azole antifungals, supporting their relevance in drug-resistance investigations. The internal laboratory Bionumbers assigned to *Candida albicans* (R3—4012545025304100), *Candida guilliermondii* (R4—4002545225304100), *Candida ciferrii* (R7—6016145637512130), and *P. aeruginosa isolates* (e.g., 0003053103500252) are also provided to facilitate future referencing. These identifiers are detailed in Supplementary File S1. The VITEK^®^ 2 identification reports are provided as Supplementary File S1.

#### 2.1.3. Selection of Multidrug-Resistant (MDR) Isolates for Gene Expression Analysis

From the 15 total isolates screened, the three most resistant strains were selected based on their antibiotic resistance profiles, as determined via VITEK^®^ 2 analysis. These isolates were chosen for a further investigation of *mexY* gene expression, a key component of the *MexXY* efflux pump, which is associated with antibiotic resistance in *P. aeruginosa* [37].

#### 2.1.4. Synthesis of Selenium Nanoparticles (SeNPs)

**Figure 1:**
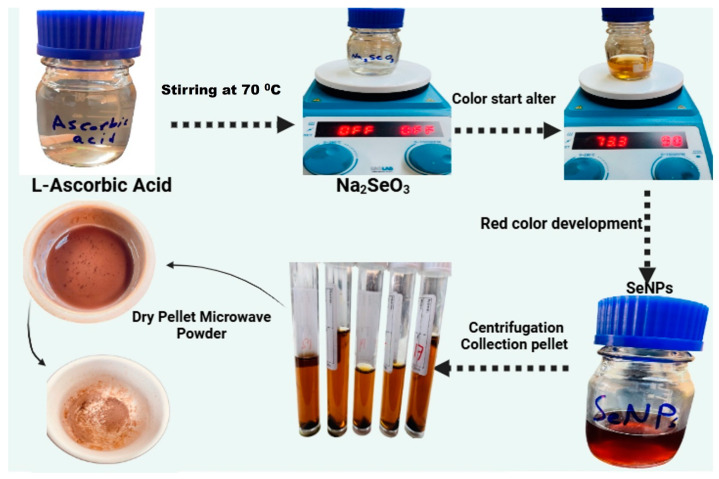
Steps involved in the synthesis of selenium nanoparticles (SeNPs).

To create SeNPs, first, 2 g of L-ascorbic acid (LAA) was mixed with 100 mL of distilled water (dH_2_O). Separately, a solution of sodium selenite (Na_2_SeO_3_Sigma Aldrich, St. Louis, MO, USA) was made by dissolving 10 mmol of the precursor salt in 100 mL of dH_2_O, heated to 70 °C. The LAA solution was then gradually added to the salt solution while continuously stirring at the same temperature. As the transparent sodium selenite solution began to turn reddish, it signaled that the synthesis of SeNPs had been successful. Afterward, the obtained product was collected and washed three times with dH_2_O via centrifugation at 4500 rpm for 15 min and oven-dried at 80 °C for 7 h. The well-dried material was then ground into a fine powder using a pastor and mortar and utilized for further characterization and bioassays. A gradual color change was observed during the reaction, transitioning from a colorless solution to a deep red hue, indicating the formation of selenium nanoparticles via reduction. Once nanoparticle synthesis was complete, centrifugation was performed to separate the SeNP precipitate from the reaction medium. The precipitate was thoroughly washed several times with deionized water after the supernatant was carefully removed to eliminate any unreacted precursors and undesirable byproducts (Figure 1). The purified selenium nanoparticle precipitate was collected and subjected to microwave-assisted drying, resulting in the formation of a fine SeNP powder suitable for further experimental applications, including MIC determinations and antifungal activity assays [38].

#### 2.1.5. Colistin-Conjugated Selenium Nanoparticles (Col-SeNPs)

**Figure 2:**
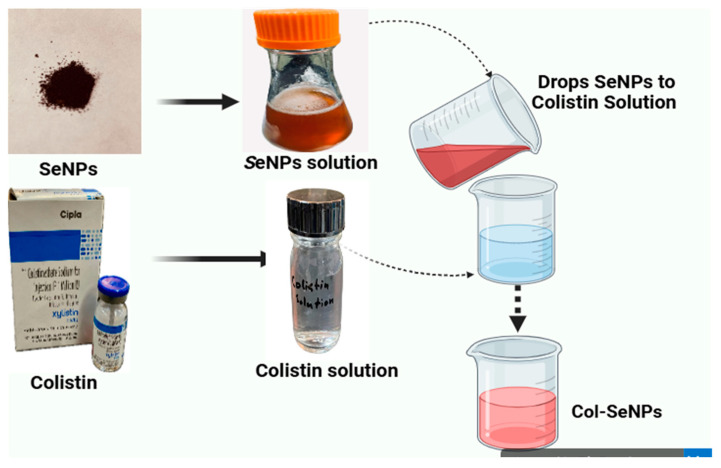
Colistin-conjugated selenium nanoparticles.

Initially, a quantity of 50 mg of selenium nanoparticles was added to a 25 mL flask that held 10 mL of distilled water. This mixture was subsequently immersed in an ultrasonic bath for 10 min at a medium setting, ensuring the complete dispersion of the nanoparticles within the distilled water. Following this, the colistin solution, with a concentration of 50 mg/L, was introduced in drops into the flask until the total volume equaled 25 mL. Subsequently, the mixture was incubated in a shaking incubator (150 rpm) for 24 h at 25 °C. The mixture was spin-dried at room temperature, and the sediment was collected in another container (Figure 2). This coating process was designed to enhance the antimicrobial efficacy and synthesis of SeNPs by increasing membrane interaction and uptake in bacterial cells [39].

### 2.2. Synthesis and Characterization of Selenium Nanoparticles (SeNPs)

#### 2.2.1. UV-Visible (UV-Vis) Spectroscopy

The analysis of the surface plasmon resonance (SPR) for biosynthesized selenium nanoparticles (SeNPs) was conducted with the aid of a PerkinElmer UV-Vis spectrophotometer (PerkinElmer, Waltham, MA, USA). An aliquot of 2 mL of synthesized SeNPs was introduced into a quartz cuvette with a path length of 1 cm, and the absorbance spectrum was obtained spanning from 200 to 800 nm. A blank reference was applied for calibration purposes [40].

#### 2.2.2. Atomic Force Microscopy (AFM)

The size distribution, three-dimensional topography, and surface morphology of the synthesized SeNPs were examined using Atomic Force Microscopy (AFM). A thin layer of SeNPs was prepared by depositing a few drops of the nanoparticle suspension onto a silica glass plate, followed by drying at room temperature in the absence of light. The dried film was then scanned using AFM to obtain topographical data. The height cumulation distribution report showed an average nanoparticle height of 10.161 nm, with the 10th percentile at 7.500 nm, median (50th percentile) at 10.000 nm, and 90th percentile at 11.250 nm, confirming uniform nanoparticle distribution [41]. The AFM analysis was conducted at the Department of Chemistry, University of Baghdad.

#### 2.2.3. Energy Dispersive X-Ray Spectroscopy (EDX)

The elemental examination of synthesized SeNPs was conducted utilizing energy dispersive X-ray spectroscopy (EDX) in combination with an electron microscope (BRUKER Q200, Berlin, Germany) [42]. This method identifies the characteristic X-ray emission fingerprints of elements to determine the elemental composition. The analysis confirmed the presence of selenium as the predominant element, with no significant contamination from other elements. The elemental mapping further verified the homogeneous distribution of selenium. The experiment was conducted at the Department of Physics, College of Sciences, University of Al-Nahrain.

#### 2.2.4. X-Ray Diffraction (XRD) Analysis

The crystalline structure and phase composition of synthesized SeNPs were investigated using X-ray diffraction (XRD) [43]. The biosynthesized selenium nanoparticles were dried and finely ground, and a thin film was prepared by dipping a cleaned glass plate into the nanoparticle suspension. The XRD pattern was recorded, and the obtained diffraction peaks were analyzed for crystallographic phase identification. The XRD study was conducted at the Al-Khura Nano-Science Center, Al Yarmouk, Baghdad, Iraq.

#### 2.2.5. Field Emission Scanning Electron Microscopy (FESEM)

The characteristics of the surface morphology and microstructure of synthesized SeNPs were analyzed using field emission scanning electron microscopy, commonly known as FESEM. This method utilizes a high-energy electron beam to create backscattered and secondary electrons, yielding high-resolution visuals of the nanoparticle surfaces. To improve conductivity, minimize charging effects, and enhance imaging resolution, the samples were coated with a thin layer of platinum, addressing the limited electron scattering in small nanoparticles. The dried SeNP suspension was sonicated in distilled water, and a small drop was placed on a glass slide and dried before platinum coating [44].

#### 2.2.6. Transmission Electron Microscopy (TEM)

The characterization of synthesized SeNPs in terms of their morphology and size distribution was conducted using transmission electron microscopy (TEM). A small drop of the SeNP suspension was placed on a TEM grid and permitted to adsorb for one minute. Following this, any surplus solution was eliminated with filter paper. Prior to imaging, the sample was dried in the air. TEM provided high-resolution structural insights, revealing the nanoparticle shape and size uniformity [45].

#### 2.2.7. Fourier Transform Infrared Spectroscopy (FTIR)

The identification of functional groups and biomolecular interactions that contribute to the stabilization of synthesized SeNPs was achieved through Fourier transform infrared spectroscopy (FTIR). A Bruker ALPHA FTIR spectrometer recorded the FTIR spectra, covering a wavenumber range from 4000 to 400 cm^−1^, with a resolution varying between 4 and 8 cm^−1^. To prepare the samples, powdered synthesized SeNPs were combined with potassium bromide (KBr) at a 1:10 ratio to form FTIR discs. The resulting spectra were plotted as % transmittance vs. wavenumber (cm^−1^) [46]. The FTIR analysis was performed at the College of Sciences, Department of Chemistry, and University of Baghdad.

#### 2.2.8. Minimum Inhibitory Concentration of SeNPs and Col-SeNPs

The broth microdilution method was employed to ascertain the minimum inhibitory concentration (MIC) of synthetic SeNPs in relation to *P. aeruginosa* strains that exhibit resistance to several drugs. SeNPs and Col-SeNPs were prepared in various concentrations (500, 250, 125, 64, and 32 μg/mL). For the inoculation of *P. aeruginosa*, initially, a base solution of SeNPs and Col-SeNPs at 500 mg/mL was created. Then, 100 microliters of this diluted solution were added to the first row of wells in a microtiter plate. Following this, each well received 100 microliters of Müller Hinton broth (MHB), with wells numbered 1 through 10. This setup utilized a two-fold dilution method, with well G serving as the positive control and well H as the negative control (MHB alone). A bacterial culture, adjusted to the McFarland standard (1.5 × 10^8^ CFU/mL), was inoculated into each well, excluding the negative control. After incubation at 37 °C for 24 h, the growth was assessed using a microtiter plate reader to measure the optical density at 450 nm. Turbid wells indicated bacterial growth, while clear wells showed no growth. The MIC is defined as the lowest concentration of synthesized SeNPs and Col-SeNPs at which no growth occurs [35].

#### 2.2.9. Antifungal Activity

The minimum inhibitory concentrations (MICs) of synthesized SeNPs (500, 250, 125, 64, and 32 μg/mL) against *Candida* spp. were also determined using the same concentrations with the microdilution method. A full night was spent cultivating each indicator strain in the proper medium. To test SeNPs, Candida species were spread thoroughly on the sterilized Petri plates with solidified Mueller–Hinton agar medium. The agar well diffusion assay was then applied. On each agar plate, five wells with a 5 mm diameter were made using a sterile cork borer, and 80 µL of different concentrations, which depend on the microdilution method, were subsequently added. The zones of inhibition were then measured the next day after being incubated for 24 h at 37 °C [45].

### 2.3. Cells, Cell Culture, and Drug Preparation

In this investigation, the MCF-7 human breast adenocarcinoma cell line was utilized. Following the protocol outlined in prior research, the cells were maintained in Roswell Park Memorial Institute1640 medium (RPMI1640) with the addition of L-glutamine (from Capricorn Scientific GmbH, Ebsdorfergrund, Germany). A complete growth medium was prepared by incorporating 10% fetal bovine serum (FBS) and a 1% solution of penicillin/streptomycin (100×; sourced from Euroclone S. p. A., Pero, Italy) into the RPMI1640 medium. The incubation took place in an environment maintained at 37 °C with 5% CO_2_ and 95% humidity, as previously described For in vitro assays, a stock solution of the compound (10 mg/mL) was created by dissolving it in DDW. The required concentrations for subsequent experiments were prepared in complete medium from the stock solution [47].

### 2.4. Cytotoxicity Assay

The evaluation of cytotoxic effects was conducted through an in vitro assay utilizing 3-(4,5-dimethylthiazol2yl)-2,5-diphenyltetrazolium bromides (MTT). A quantity of 7000 cells was plated in every well of a 96-well plate and left to incubate overnight to promote cell attachment. Cell lines (MCF-7) were then exposed to increasing concentrations of compounds (100–500 μg/mL), and three replicate wells were used for each treatment. Following incubation (24 h), the medium was removed from the plate, and 20 μL of the MTT solution (5 mg/mL) (Shanghai Macklin Biochemical Co., Ltd., Shanghai, China) was added to each well and incubated for 3 h at 37 °C in the dark. To prepare the MTT solution, 50 μL of DMSO from Bio Basic Inc. (Markham, ON, Canada) was added and then shaken for 10 min. Absorbance readings were taken at 490 nm using a microplate reader from BioTek Instruments, Inc. (Winooski, VT, USA). To calculate the percentage of viable cells based on the raw absorbance values, the following formula was employed: viability% = (A_test_ − A_blank_)/(A_control_ − A_blank_) × 100, with ‘A’ indicating absorbance. A dose–response curve was created utilizing GraphPad Prism software version 6 from Dotmatics (Boston, MA, USA), which also allowed for the determination of the growth inhibitory concentration that decreases cell viability by 50% (GI50) [48].

### 2.5. RT-qPCR Protocol

#### RNA Purification

RNA was extracted from samples following the TRIzol™ Reagent protocol, which includes the steps outlined below:

- Sample Lysis

For cells grown in suspension, the culture underwent centrifugation for two minutes at a speed of 13,000 rpm. After discarding the supernatant, 0.5 mL of TRIzol™ Reagent was introduced to the pellet. The lysate was subsequently homogenized through repeated pipetting.

- Three-Phase Separation

To each tube, 0.2 mL of chloroform was added, and the tube was securely capped. After incubating for two to three minutes, the mixtures were centrifuged for ten minutes at 12,000 rpm, yielding three separate layers: a bottom organic layer, an interphase, and a transparent top aqueous layer. The RNA containing aqueous layer was then moved to a new tube.

- RNA Precipitation

The mixture of the aqueous phase and 5 mL of isopropanol was incubated for ten minutes, followed by centrifugation at 12,000 rpm for an additional ten minutes. After the total RNA had formed a precipitate, the supernatant was removed, resulting in a white gel-like pellet remaining at the tube’s bottom.

### 2.6. Detection of fbp Gene: DNA Extraction

Genomic DNA was isolated from bacterial cultures following the ABIO pure extraction protocol, as detailed in Table 1 [49].

### 2.7. Primer Preparation

The Macrogen Company supplied the primers in a lyophilized state. To prepare a stock solution, the primers were reconstituted in nuclease-free water, resulting in a concentration of 100 pmol/μL. To create a working primer solution of 10 pmol/μL, 10 μL from the stock was combined with 90 μL of nuclease-free water and then kept at 20 °C.

### 2.8. Agarose Gel Electrophoresis

Following the PCR amplification, agarose gel electrophoresis was performed to verify the presence of the amplified products. This PCR procedure depended on the specifications of the isolated DNA. Each PCR product was promptly loaded, with 5 μL from each sample being directly inserted into the wells. The gel was then operated at a voltage of 100 V for a duration of 60 min, facilitating the movement of DNA from the cathode towards the anode. Bands stained with ethidium bromide within the gel were observed through gel-imaging technology.

### 2.9. RNA Purification

RNA was isolated from samples according to the TRIzol™ Reagent protocol.

### 2.10. Determination of RNA and cDNA Yield

To assess the concentration of the extracted RNA and evaluate sample quality for further applications, a Quantus Fluorometer was used. For the measurement, 1 µL of RNA was mixed with 200 µL of diluted Quanti Fluor Dye. After incubating for 5 min at room temperature in the dark, the RNA concentration was measured.

### 2.11. Ethical Statement

The Committee of Ethical Standards at the College of Sciences, Baghdad University, granted approval for this research. A local ethics committee evaluated and sanctioned the study protocol, the information provided to subjects, and the consent form, with the document numbered FM. SA/308, dated 25 December 2023.

### 2.12. Statistical Analysis

A one-way analysis of variance (ANOVA) using Tukey’s method along with a Student’s test was conducted to determine the significance of group variance. Statistical significance was considered at *p* < 0.05 or *p* < 0.01. Each sample that underwent statistical analysis was tested in triplicate. The data were presented as the mean ± standard deviation, and the statistical analyses were performed with GraphPad Prism version 9 (GraphPad Software Inc., La Jolla, CA, USA).

## 3. Results

### 3.1. Identification of Pseudomonas aeruginosa

**Figure 3:**
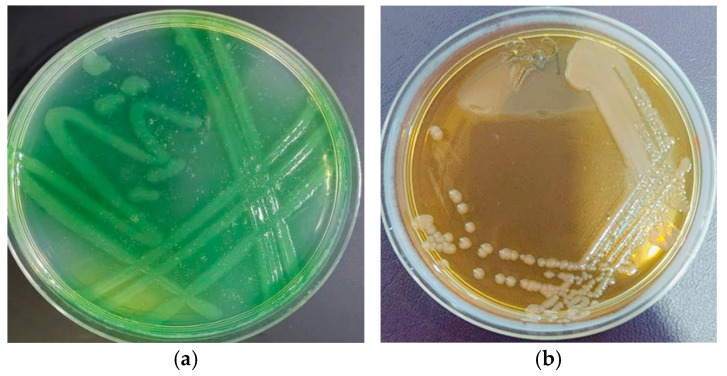
*P. aeruginosa* on (**a**) MacConkey agar and (**b**) Cetrimide agar.

The colonies exhibited a pale yellow color with a sweaty grape aroma on MacConkey agar, while they presented a greenish yellow hue on the selective Pseudomonas Cetrimide medium (Figure 3). The identification of *Pseudomonas aeruginosa* isolates was conducted using an automated bioMérieux VITEK^®^ 2 Compact system, which employs biochemical profiling and probabilistic algorithms for microbial identification. Multiple clinical and environmental isolates were analyzed, and the system generated results within 4.85–5.95 h, providing high-confidence identifications of *P. aeruginosa* with probabilities ranging from 95% to 99%.

The biochemical profile associated with *P. aeruginosa* included positive oxidase activity, the ability to grow at 42 °C, and resistance to a range of β-lactam antibiotics. The VITEK^®^ 2 system assigned distinct bio numbers to each isolate, confirming the species-level identification.

### 3.2. Antimicrobial Susceptibility Testing (AST)

**Figure 4:**
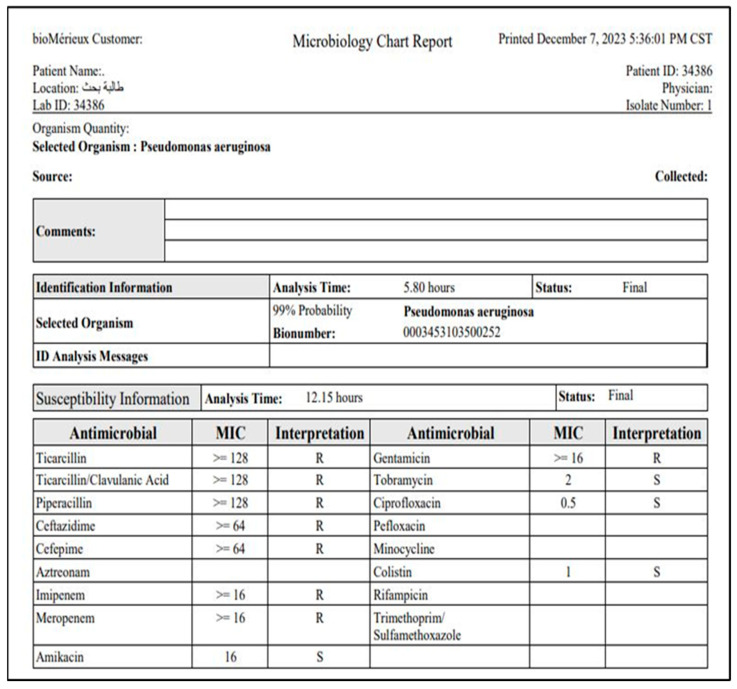
Identification of *P. aeruginosa* using VITEK 2 compact system.

Antimicrobial susceptibility testing (AST) was conducted with the VITEK^®^ 2 ASTGN card, which assesses the minimum inhibitory concentration (MIC) across various antibiotics. The AST results demonstrated significant variability in resistance patterns among isolates (Figure 4).

### 3.3. Multidrug Resistance Patterns

Β-lactam resistance: A subset of isolates exhibited resistance to Ticarcillin (MIC ≥ 64 μg/mL) and Piperacillin (MIC ≥ 64 μg/mL), with some strains resistant to Ceftazidime (MIC ≥ 64 μg/mL) and Cefepime (MIC ≥ 64 μg/mL). Carbapenem resistance: Several isolates displayed elevated MICs for Imipenem and Meropenem (≥16 μg/mL), indicating carbapenem-resistant *P. aeruginosa* (CRPA). Aminoglycoside susceptibility: Most isolates were susceptible to Amikacin (MIC ≤ 2 μg/mL), Gentamicin (MIC ≤ 1–2 μg/mL), and Tobramycin (MIC ≤ 1 μg/mL). Fluoroquinolone resistance: Resistance was observed in some isolates, with Ciprofloxacin MIC values ≥ 0.5 μg/mL. However, the majority of isolates remained susceptible (MIC ≤ 0.25 μg/mL). Colistin susceptibility: all isolates demonstrated susceptibility to Colistin (MIC = 1–2 μg/mL), suggesting that Colistin remains an effective treatment option for drug-resistant *P. aeruginosa*.

### 3.4. Clinical Significance

The identification and resistance profiling of *Pseudomonas aeruginosa* isolates highlight the emergence of multidrug-resistant (MDR) and carbapenem-resistant strains, which pose significant challenges in clinical management. The high susceptibility to Colistin and aminoglycosides suggests their potential role in combination therapy for resistant infections. The results emphasize the necessity for continuous surveillance and antimicrobial stewardship to mitigate the spread of resistant *P. aeruginosa* strains.

### 3.5. Characterization of Biosynthesized SeNPs

#### 3.5.1. UV-Visible (UV-VIS) Spectroscopy

**Figure 5:**
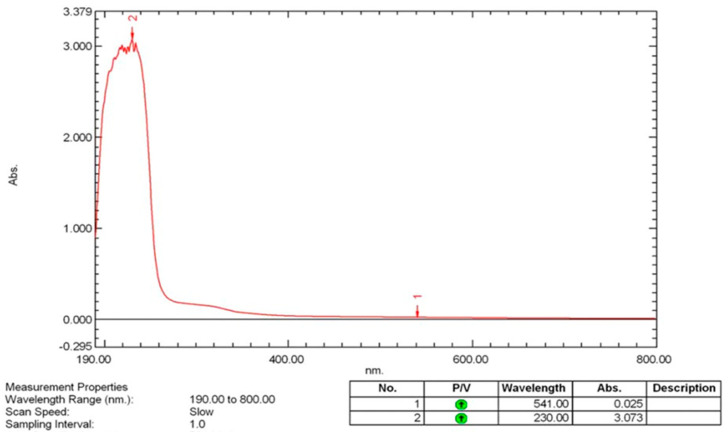
UV–vis spectroscopy analysis of synthesized SeNPs.

UV-Vis spectroscopy was employed to determine the optical properties and confirm the formation of biosynthesized selenium nanoparticles (SeNPs). The absorbance spectrum was recorded over a wavelength range of 200–800 nm using a PerkinElmer spectrophotometer, with deionized water serving as the reference blank. A well-defined absorption peak was observed, characteristic of the surface plasmon resonance (SPR) of synthesized SeNPs. The presence of this peak strongly indicates the formation of selenium nanoparticles, as SPR results from the collective oscillation of conduction-band electrons in response to incident light. The peak position and intensity suggest the formation of stable synthesized SeNPs with well-defined electronic properties (Figure 5). The absence of additional peaks corresponding to bulk selenium or selenium oxides confirms the high purity of the synthesized nanoparticles, with no significant oxidation or aggregation.

#### 3.5.2. Atomic Force Microscopy (AFM)

**Figure 6:**
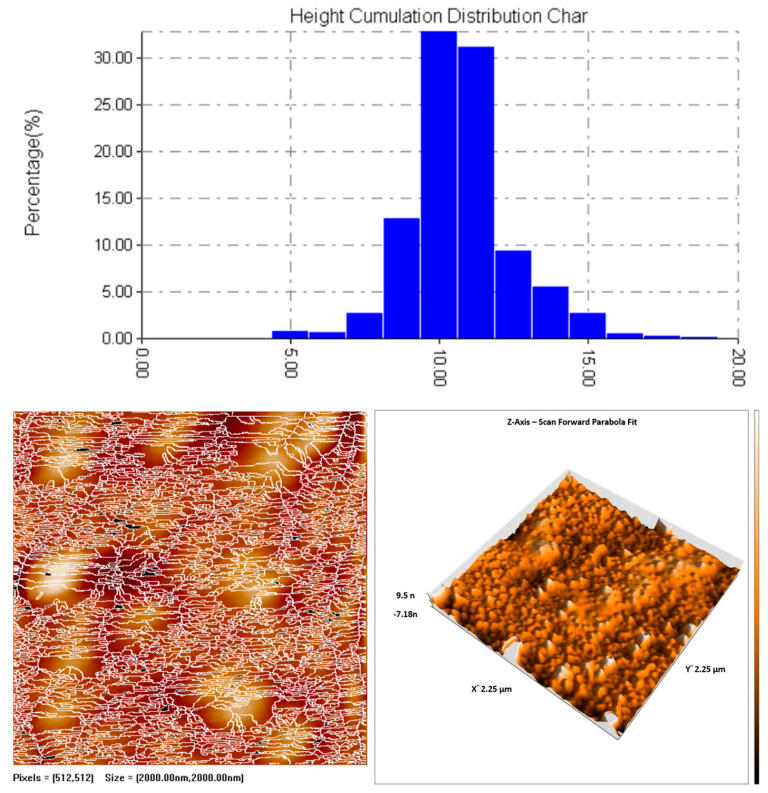
Three-dimensional AFM images of synthesized SeNPs.

The topographical characteristics and size distribution of synthesized SeNPs were analyzed using atomic force microscopy (AFM). The nanoparticles were deposited onto a silica glass substrate and allowed to dry under ambient conditions to ensure minimal interference from external factors. The AFM height cumulation distribution report indicated an average particle height of 10.161 nm, with the 10th percentile at 7.500 nm, the median (50th percentile) at 10.000 nm, and the 90th percentile at 11.250 nm Figure 6. The relatively narrow distribution suggests a high degree of uniformity in nanoparticle synthesis, indicative of controlled nucleation and growth processes. The three-dimensional AFM images confirmed the presence of discrete, well-defined nanoparticles without significant aggregation, supporting the findings from electron microscopy techniques.

#### 3.5.3. Energy Dispersive X-Ray Spectroscopy (EDX) Analysis of Selenium Nanoparticles (SeNPs)

**Figure 7:**
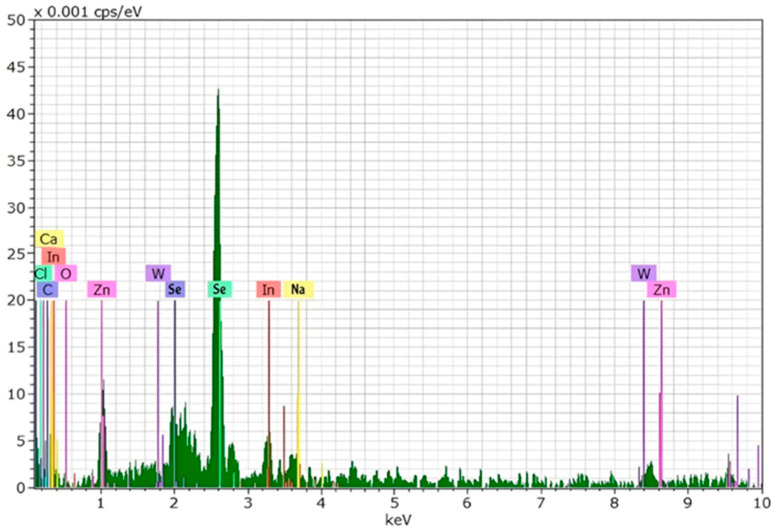
EDX of synthesis of SeNPs.

The energy dispersive X-ray spectroscopy (EDX) spectrum of the synthesized selenium nanoparticles (SeNPs) provides a detailed elemental composition of the sample, confirming the presence of selenium as the primary component. The spectrum exhibits prominent peaks corresponding to selenium (Se), particularly at approximately 1.4 keV and 3.8 keV, which are characteristic of selenium’s Lα and Kα emission lines, respectively. These peaks strongly indicate the successful synthesis of selenium nanoparticles (Figure 7).

In addition to selenium, the EDX spectrum reveals the presence of several other elements, including oxygen (O), carbon (C), chlorine (Cl), calcium (Ca), zinc (Zn), tungsten (W), indium (In), and sodium (Na). The presence of oxygen suggests the possible oxidation of selenium nanoparticles, which is a common occurrence due to exposure to atmospheric oxygen or interactions with the stabilizing agents used during synthesis. Carbon and chlorine may be attributed to organic or inorganic stabilizing agents, surfactants, or precursor materials used in the nanoparticle-synthesis process.

The detection of calcium (Ca), zinc (Zn), tungsten (W), indium (In), and sodium (Na) in trace amounts could be due to impurities from the synthesis reagents, the reaction environment, or possible adsorption of these elements onto the surface of the nanoparticles. These trace elements may also be present due to interactions with the substrate or residual compounds from the synthesis medium.

The overall elemental profile confirms that selenium is the dominant component, validating the successful formation of selenium nanoparticles. The presence of minor elements provides insights into the synthesis conditions and potential sources of impurities, which may influence the physicochemical properties of the nanoparticles. Further purification steps or characterization techniques, such as X-ray diffraction (XRD) and Fourier-transform infrared spectroscopy (FTIR), may be required to confirm the crystalline nature, phase purity, and functional group interactions of the synthesized SeNPs.

#### 3.5.4. X-Ray Diffraction (XRD) Analysis

**Figure 8:**
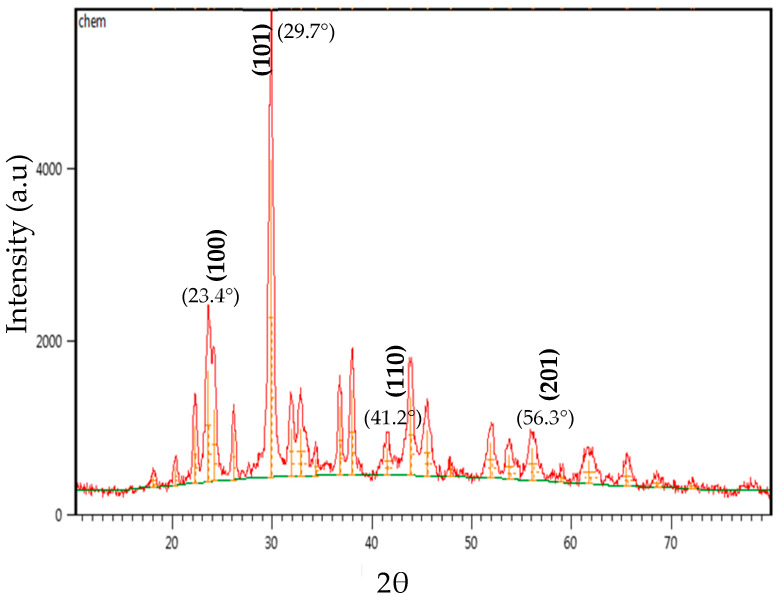
XRD of synthesized SeNPs.

The crystallinity and phase composition of the biosynthesized SeNPs were analyzed using X-ray diffraction (XRD). The diffraction pattern exhibited distinct peaks corresponding to the hexagonal crystalline phase of selenium (JCPDS Card No. 06-0362). The observed diffraction peaks at 2θ = ~23.4°, 29.7°, 41.2°, and 56.3° correspond to the (100), (101), (110), and (201) planes, respectively, confirming the presence of nanocrystalline selenium (Figure 8). The broadening of the diffraction peaks is indicative of nanoscale crystallite sizes, as per the Scherrer equation, suggesting that the synthesis of synthesized SeNPs exhibit a high surface-area-to-volume ratio. The absence of peaks corresponding to selenium dioxide (SeO_2_) or amorphous selenium confirms that the synthesized nanoparticles maintain their elemental composition without oxidation.

#### 3.5.5. Field Emission Scanning Electron Microscopy (FESEM)

**Figure 9:**
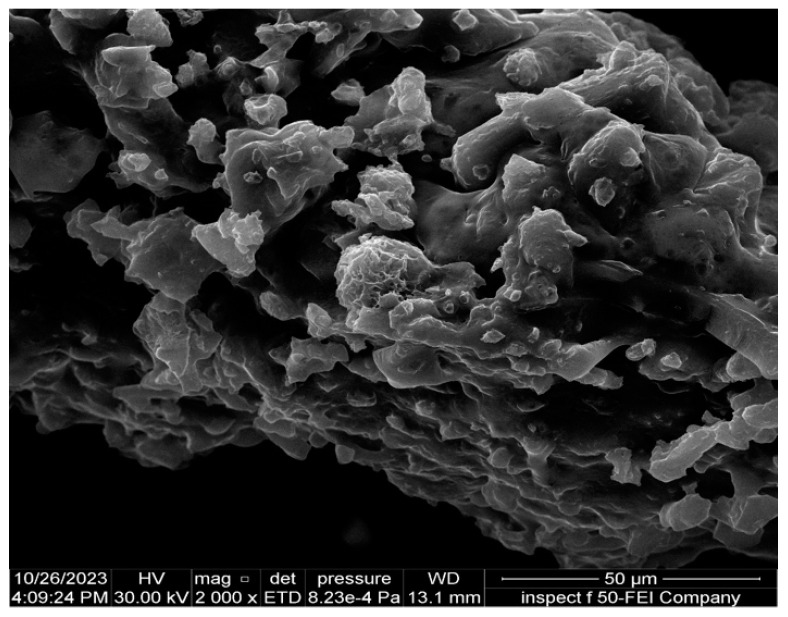
SEM images of morphology of the SeNPs at a scale bar of 50 nm.

The features of synthesized SeNPs at the morphological level were analyzed through field emission scanning electron microscopy or FESEM, known for its ability to deliver high-resolution images of nanoparticle surfaces. The micrographs revealed that the selenium nanoparticles exhibited a predominantly spherical morphology with minimal aggregation (Figure 9). The nanoparticles appeared to be well-separated, suggesting that the biosynthetic method facilitated the stabilization of individual particles, likely due to the presence of biomolecules acting as capping agents. A thin platinum coating was applied to the sample to enhance conductivity and prevent electron beam-induced surface charging effects. The obtained images demonstrated uniform nanoparticle distribution with smooth surfaces, further supporting the results from AFM and XRD. The consistency in size and morphology underscores the reproducibility of the biosynthetic process.

#### 3.5.6. Transmission Electron Microscopy (TEM)

**Figure 10:**
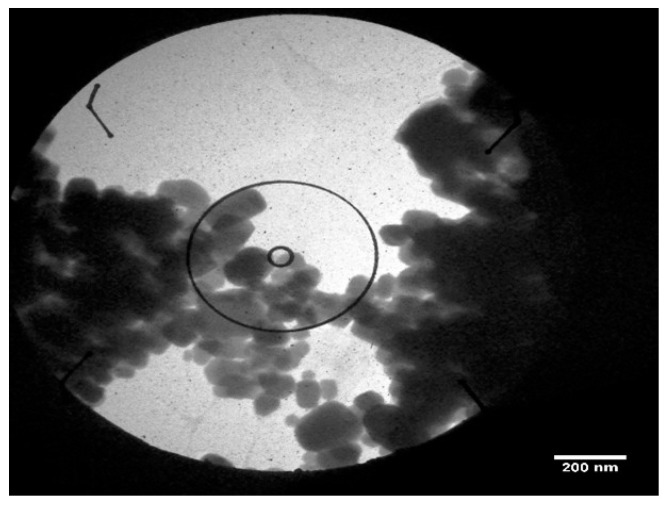
Transmission electron microscopy (TEM) image of selenium nanoparticles (SeNPs) showing a clustered spherical morphology.

To achieve higher-resolution structural analysis, transmission electron microscopy (TEM) was used to examine the internal structure and precise size distribution of synthesized SeNPs. The TEM (Figure 10) revealed that the nanoparticles exhibited a uniform spherical shape, with well-defined boundaries, confirming their monodisperse nature. The particles showed an average diameter within the 10–15 nm range, consistent with AFM and FESEM findings. The high contrast of the TEM images suggests that the nanoparticles have a dense core, indicative of a crystalline structure. The absence of significant aggregation further supports the hypothesis that biological molecules present in the synthesis medium act as stabilizing agents.

#### 3.5.7. Fourier Transform Infrared Spectroscopy (FTIR)

**Figure 11:**
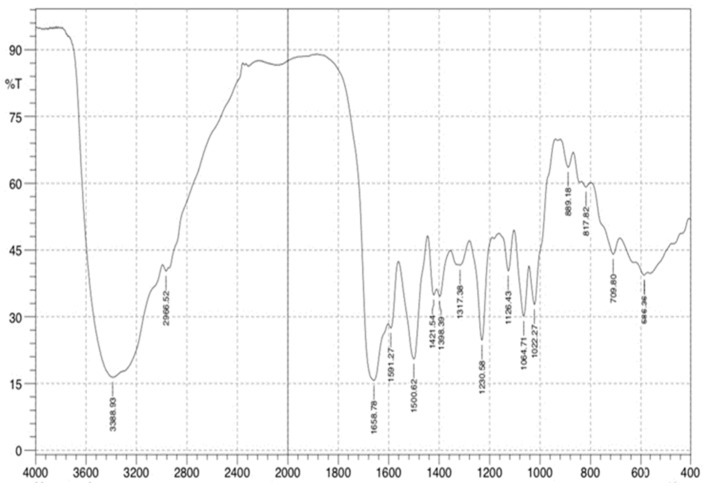
FTIR analysis of antimicrobial activity of SeNPs and Col-SeNP sagainst clinical isolates of *Pseudomonas aeruginosa*.

FTIR spectroscopy was conducted to identify functional groups associated with the synthesis of SeNPs and determine the possible biomolecular interactions responsible for their synthesis and stabilization. The spectrum displayed characteristic absorption bands corresponding to various organic functional groups: (i) A broad peak around 3300–3500 cm^−1^, attributed to hydroxyl (-OH) and amine (-NH) stretching vibrations, suggesting the presence of proteins or polysaccharides involved in nanoparticle stabilization; (ii) peaks in the 1650–1750 cm^−1^ region, corresponding to C=O stretching of amide and carbonyl groups, indicating interactions with organic molecules that may act as capping agents; (iii) peaks at 1400–1500 cm^−1^, characteristic of C=C stretching vibrations, suggest the presence of aromatic compounds involved in nanoparticle stabilization; (iv) absorption bands in the 1000–1100 cm^−1^ range, indicative of C-O stretching (Figure 11), which could be attributed to polysaccharides or phenolic compounds. The FTIR results confirm that biomolecules present in the synthesis medium play a crucial role in the reduction, stabilization, and capping of synthesized SeNPs, preventing aggregation and enhancing their dispersibility in aqueous media.

**Figure 12:**
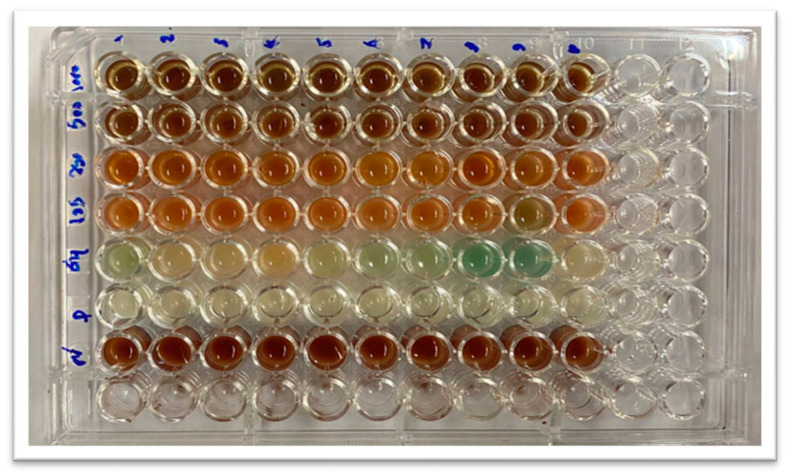
MICs of different concentrations (32, 64, 125, 250, 500) μg/mL of SeNPs and Col-SeNPs against multidrug-resistant *P. aeruginosa*.

This research is focused on examining the antibacterial effects of SeNPs and Col-SeNPs nanoparticles, with a particular emphasis on their physicochemical traits and possible mechanisms by which they operate. Additionally, the discussion will extend to encompass the precursors, various synthesis techniques, dimensions, configurations, concentrations, and surface modifications that impact the antibacterial effectiveness of nanoparticles. To determine the minimum inhibitory concentration (MIC), a microdilution broth approach was utilized in a sterile 96-well microplate, as shown in Figure 12. For these tests, a bacterial culture was prepared and incubated for 24 h. A 0.5 McFarland standard bacterial suspension was formed in Müller–Hinton broth and introduced into all wells of the plate. The MIC for synthesized SeNPs and Col-SeNPs was identified as the minimum concentration without any visible growth of bacteria, which was found to be 125 μg/mL across all ten multidrug-resistant *P. Aeruginosa* isolates. The subMIC was set at 64 μg/mL. The distinctive antibacterial features of synthesized SeNPs and Col-SeNPs stem from their unique physical and chemical properties, such as the surface charge and capability to produce reactive oxygen species (ROS). Factors that affect the characteristics of nanoparticles include the chosen synthesis method and selection of precursors, as well as their size, shape, and concentrations.

#### 3.5.8. Determination of Minimum Inhibitory Concentration (MIC) of SeNPs Against *Candida* spp.

The antifungal properties of selenium nanoparticles (SeNPs) were assessed against Candida species using the agar well diffusion technique and the broth microdilution test to ascertain the minimum inhibitory concentration (MIC). Five distinct concentrations of SeNPs (500, 250, 125, 64, and 32 μg/mL) were examined, and the impact on fungal proliferation was evaluated by observing the development of clear inhibition zones around the wells following 24 h of incubation at 37 °C.

#### 3.5.9. Zone of Inhibition Analysis

**Figure 13:**
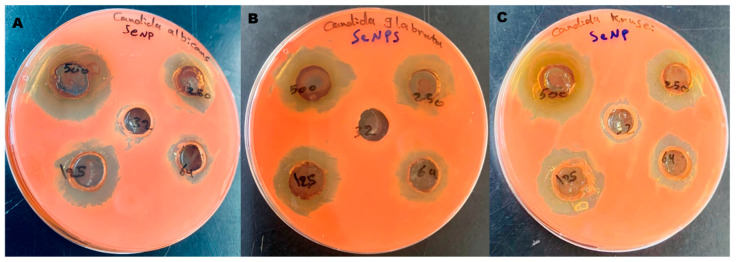
Antifungal activity of selenium nanoparticles (SeNPs) against (**A**) *Candida albicans*, (**B**) *Candida glabrata*, and (**C**) *Candida krusei*. The agar well diffusion method was used, and inhibition zones are visible at various concentrations of SeNPs (32, 64, 125, 250, and 500 µg/mL).

Figure 13 shows the antifungal activity of selenium nanoparticles (SeNPs) against (A) *Candida albicans*, (B) *Candida glabrata*, and (C) *Candida krusei*. The agar well diffusion method was used, and inhibition zones are visible at various concentrations of SeNPs (32, 64, 125, 250, and 500 µg/mL). The results demonstrated a dose-dependent antifungal effect, with increasing concentrations of synthesized SeNPs correlating with larger inhibition zones. The highest concentration (500 μg/mL) exhibited the largest inhibition zone, whereas lower concentrations (64 and 32 μg/mL) displayed minimal or no inhibition, indicating reduced antifungal activity at suboptimal doses.

#### 3.5.10. MIC Determination Using Broth Microdilution

The MIC values were determined by monitoring fungal growth inhibition in microtiter plates after incubation with SeNPs. The lowest concentration at which no visible fungal growth was observed was designated as the MIC. The results showed that the MIC for SeNPs against *Candida* spp. ranged between 125 and 250 μg/mL, depending on the strain tested. These findings indicate that synthesized SeNPs exhibit significant antifungal activity, with potential for use as an alternative or adjunct antifungal agent.

#### 3.5.11. Antifungal Activity of Selenium Nanoparticles and Chitosan-Functionalized Selenium Nanoparticles

**Figure 14:**
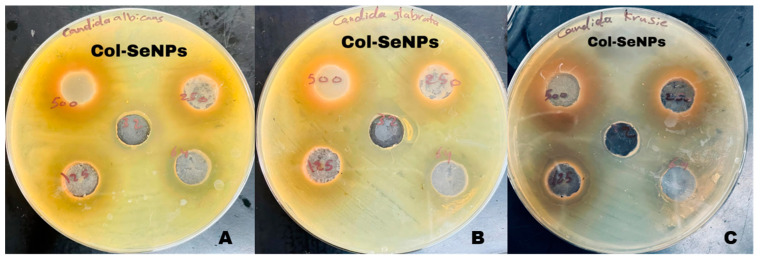
Antifungal activity of colistin-conjugated selenium nanoparticles (Col-SeNPs) against (**A**) *Candida albicans*, (**B**) *Candida glabrata*, and (**C**) *Candida krusei*. Inhibition zones were observed at concentrations of 32, 64, 125, 250, and 500 µg/mL using the agar well diffusion method.

The antifungal activity of selenium nanoparticles (SeNPs) and chitosan-functionalized selenium nanoparticles (Col-SeNPs) was evaluated against *Candida glabrata*, *Candida krusei*, and *Candida albicans* at concentrations of 32, 64, 125, 250, and 500 µg/mL. The results demonstrated a clear concentration-dependent increase in inhibition zones for both treatments, with Col-SeNPs consistently exhibiting superior antifungal efficacy across all tested strains. A statistical analysis using Tukey’s multiple comparisons test confirmed significant differences between treatment groups, particularly at higher concentrations. Figure 14 presents representative antifungal susceptibility images for Col-SeNPs, showing pronounced inhibition zones for all three *Candida* species, further confirming their enhanced antifungal performance relative to SeNPs alone.

**Figure 15:**
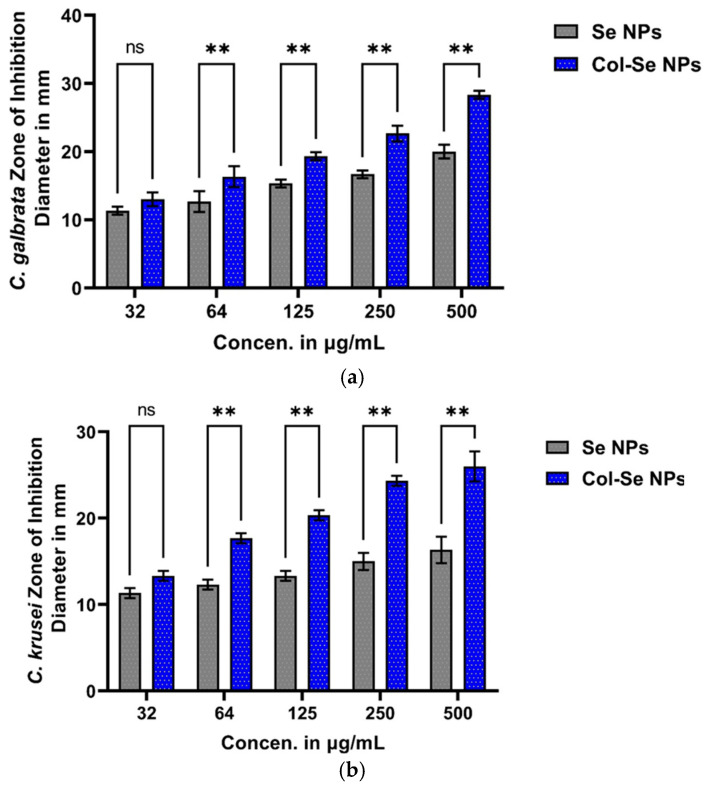

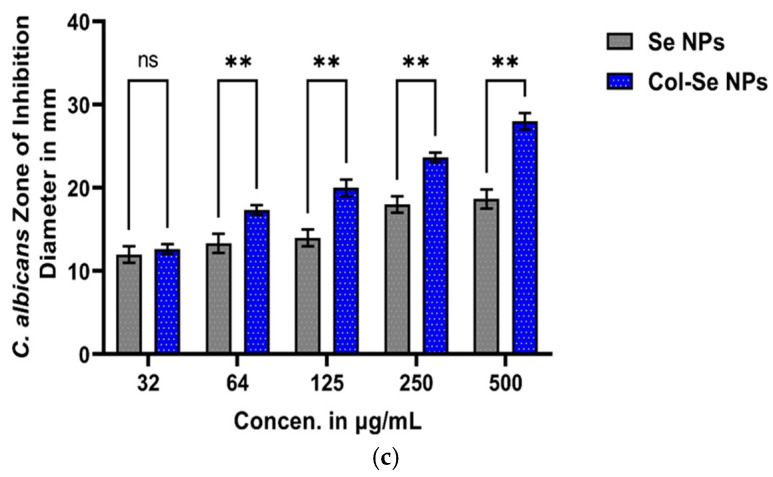
Comparative antifungal activity of selenium nanoparticles (SeNPs) and colistin-conjugated selenium nanoparticles (Col-SeNPs) against *Candida* spp. measured by zone of inhibition diameter (mm) at different concentrations (32–500 µg/mL): (**a**) *Candida glabrata*, (**b**) *Candida krusei*, (**c**) *Candida albicans*. Data represent mean ± SD; ** *p* < 0.001, ns = not significant (Tukey’s test).

For *Candida glabrata,* inhibition zones increased with the concentration for both nanoparticles. At the lowest concentration (32 µg/mL), SeNPs exhibited an inhibition zone of 11.33 ± 0.6 mm, whereas Col-SeNPs showed a slightly higher inhibition of 13.0 ± 1.0 mm, though the difference was not statistically significant (*p* = 0.2302). However, from 64 µg/mL onwards, Col-SeNPs demonstrated significantly greater antifungal activity compared to SeNPs (*p* < 0.01). At the highest concentration (500 µg/mL), the inhibition zone for SeNPs reached 20.0 ± 1.0 mm, whereas Col-SeNPs exhibited a significantly larger inhibition zone of 28.30 ± 0.6 mm (*p* < 0.0001). Tukey’s post hoc test further confirmed significant differences in inhibition across different concentrations for both nanoparticles, particularly between the lower and higher concentrations show Figure 15a.

In the case of Candida krusei, Col-SeNPs displayed markedly higher antifungal efficacy than SeNPs at concentrations above 64 µg/mL. At 32 µg/mL, SeNPs and Col-SeNPs showed inhibition zones of 11.33 ± 0.6 mm and 13.33 ± 0.6 mm, respectively, but the difference was not statistically significant (*p* = 0.0775). However, at 64 µg/mL and above, the difference became highly significant (*p* < 0.0001), with Col-SeNPs consistently producing larger inhibition zones. At the highest concentration (500 µg/mL), SeNPs exhibited an inhibition zone of 16.33 ± 1.5 mm, whereas Col-SeNPs reached 26.0 ± 1.7 mm (*p* < 0.0001). Tukey’s analysis revealed non-significant differences between some lower concentrations for SeNPs, while Col-SeNPs showed statistically significant inhibition fornearly all concentration comparisons (Figure 15b).

Similarly, *Candida albicans* exhibited a dose-dependent response to both nanoparticles, with Col-SeNPs showing greater antifungal activity. At 32 µg/mL, the inhibition zone was 12.0 ± 1.0 mm for SeNPs and 12.67 ± 0.6 mm for Col-SeNPs, with no significant difference (*p* = 0.9161). However, at 64 µg/mL, the difference became significant (*p* = 0.0002), with inhibition zones of 13.33 ± 1.2 mm for SeNPs and 17.33 ± 0.6 mm for Col-SeNPs. At the highest concentration (500 µg/mL), SeNPs produced an inhibition zone of 18.7 ± 1.2 mm, whereas Col-SeNPs showed a significantly higher inhibition zone of 28.0 ± 1.0 mm (*p* < 0.0001). Tukey’s multiple comparisons test revealed that while SeNPs exhibited non-significant differences at lower concentrations, Col-SeNPs displayed significant inhibition differences across all concentration comparisons (Figure 15c).

Overall, these findings demonstrate that Col-SeNPs possess significantly enhanced antifungal activity compared to SeNPs, suggesting the beneficial role of chitosan functionalization in improving antifungal efficacy. The observed dose-dependent increase in inhibition highlights the potential of Col-SeNPs as an effective antifungal agent, particularly at higher concentrations. The statistical validation further confirms the superior performance of Col-SeNPs, making them a promising candidate for antifungal applications.

### 3.6. Cytotoxicity Assessment of the Compound on MCF-7 Cells

#### 3.6.1. Cell Viability and Dose-Dependent Cytotoxic Effects

**Figure 16:**
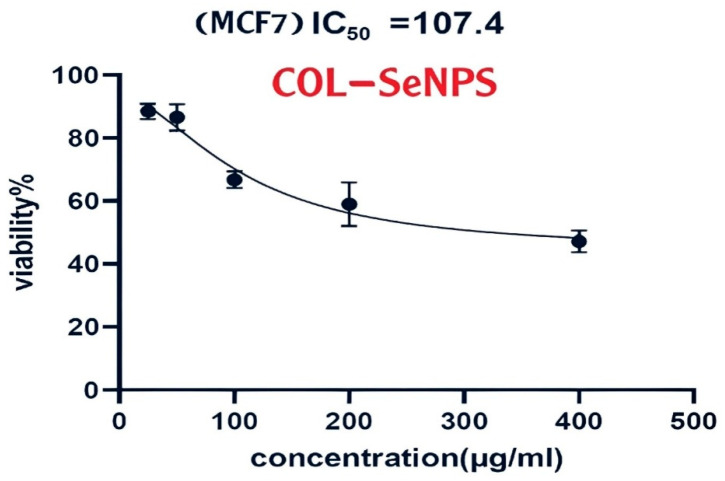

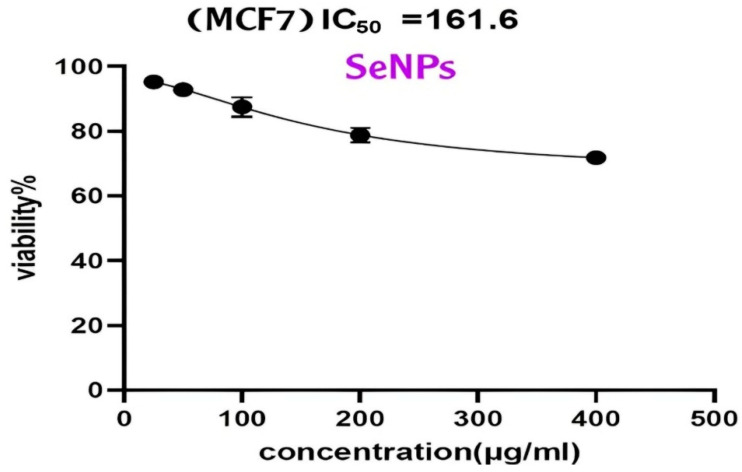
Cytotoxic effects of SeNPs and Col-SeNPs on MCF-7 cells.

The evaluation of the cytotoxic impact of the investigated compound on the human breast adenocarcinoma cell line (MCF-7) was conducted using the MTT assay, which stands for 3-(4,5-dimethylthiazol2yl)-2,5-diphenyltetrazolium bromide. For 24 h, MCF-7 cells underwent treatment with escalating concentrations of the compound ranging from 100 to 500 μg/mL, and the viability percentage of the cells was determined in comparison to the untreated control group (Figure 16). The viability of MCF-7 cells exhibited a dose-dependent decline upon compound exposure. At the lowest concentration (100 μg/mL), a moderate reduction in cell viability was observed, whereas at the highest tested concentration (500 μg/mL), a substantial decrease in cell survival was evident. The half-maximal growth inhibitory concentration (GI50) was determined using nonlinear regression analysis of the dose–response curve, as generated using GraphPad Prism (version 6, Dotmatics). The calculated GI50 value indicates the effective concentration required to reduce cell viability by 50%, which serves as a key parameter in evaluating the compound’s cytotoxic potency.

The MTT assay results demonstrated dose-dependent cytotoxicity of both SeNPs and Col-SeNPs in MCF-7 cells. Notably, Col-SeNPs exhibited a lower IC_50_ value compared to SeNPs, indicating enhanced anticancer activity. This increased potency may be attributed to a synergistic effect between colistin and selenium nanoparticles. Colistin is known to increase cell membrane permeability, which may facilitate the more efficient internalization of SeNPs into cancer cells. Additionally, the conjugated system may enhance oxidative stress and promote apoptosis more effectively than SeNPs alone. These findings support the potential of Col-SeNPs as a more potent therapeutic agent against breast cancer cells and justify further investigation into their mechanism of action.

#### 3.6.2. Statistical Analysis and Interpretation

In order to verify the reliability of the results, each experiment was conducted three times (n = 3) under the same conditions, with the data represented as the mean ± standard deviation (SD). A statistically significant reduction in cell viability (*p* < 0.05) was noted at elevated concentrations of the compound, validating its cytotoxic effects on MCF-7 cells. The results suggest that the compound exerts an inhibitory effect on cell proliferation in a dose-dependent manner, consistent with its potential role as an anticancer agent.

#### 3.6.3. Mechanistic Implications and Future Considerations

The observed cytotoxicity suggests that the tested compound interferes with essential cellular processes, leading to impaired metabolic activity and reduced proliferation in MCF-7 cells. The exact mechanism underlying this effect remains to be elucidated but may involve apoptotic induction, oxidative stress, or the disruption of cell cycle progression, which warrants further investigation. Future studies will focus on mechanistic assays, including flow cytometry for apoptosis detection, reactive oxygen species (ROS) quantification, and cell cycle analysis, to delineate the molecular pathways involved.

#### 3.6.4. Gene Expression Analysis of *mexY* in Response to Treatments

The gene expression analysis of *mexY* was assessed in response to different treatments, including colistin, selenium nanoparticles (SeNPs), and colistin-functionalized selenium nanoparticles (Col-SeNPs), using quantitative real-time PCR (qRT-PCR). Expression levels were compared against an untreated control group, with results presented as fold-changes relative to the control (2^−ΔΔCt^). As shown in Figure 15, no statistically significant differences in *mexY* expression were observed between SeNP and Col-SeNP treatments for isolates A and C. This could be attributed to isolate-specific resistance mechanisms. These particular strains may harbor stable genetic alterations—such as plasmid-borne *mexY* overexpression or chromosomal mutations—that limit the ability of either treatment to effectively downregulate efflux pump expression. In contrast, isolate B showed a more pronounced response, indicating variability in susceptibility among strains. These findings suggest that the impact of Col-SeNPs on efflux pump modulation may depend on the underlying resistance profile of each isolate.

**Figure 17:**
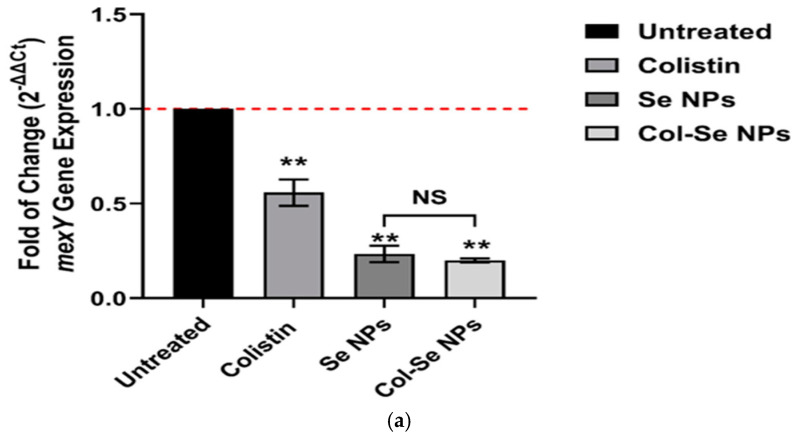

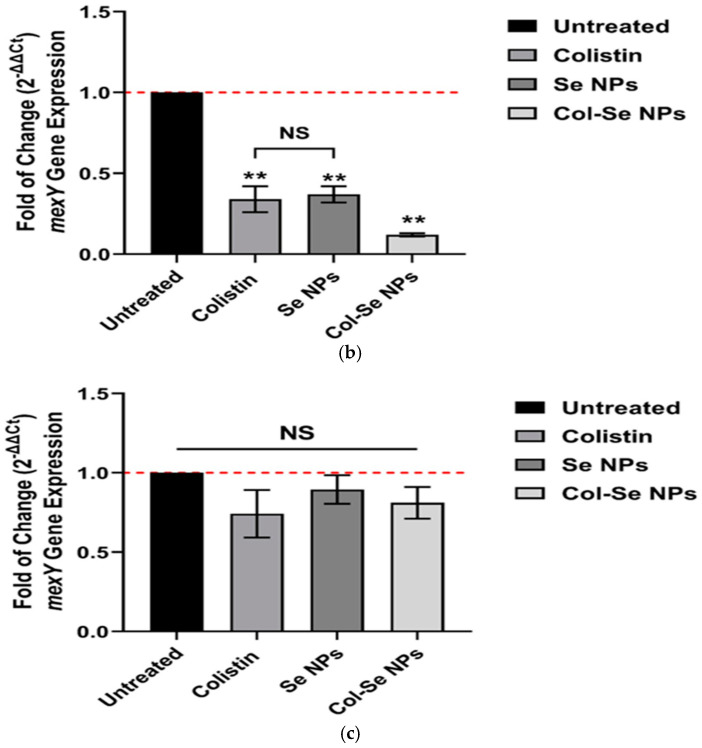
Fold-change in *mexY* gene expression in three *Pseudomonas aeruginosa* isolates (**a**–**c**) after treatment with colistin, selenium nanoparticles (SeNPs), and colistin-conjugated selenium nanoparticles (Col-SeNPs), measured via qRT-PCR. Results are presented as the mean ± standard deviation (SD), n = 3. Fold-changes are calculated using the 2^−ΔΔCt^ method and normalized to the untreated control (set as 1.0). Statistical comparisons were made using one-way ANOVA followed by Tukey’s post hoc test. ** *p* < 0.05; ns = not significant, SD: standard deviation.

In Figure 17a, the expression of *mexY* was significantly downregulated in response to SeNPs and Col-SeNPs compared to the untreated control (*p* < 0.01). Colistin treatment also led to a significant reduction in *mexY* expression, though to a lesser extent than SeNPs and Col-SeNPs. No statistically significant difference (NS) was observed between the SeNP and Col-SeNP groups, suggesting that colistin functionalization did not further enhance the suppression of *mexY* expression.

In Figure 17b, colistin and SeNPs both resulted in the significant downregulation of *mexY* expression compared to the untreated group (*p* < 0.01). However, the most pronounced reduction in *mexY* expression was observed in the Col-SeNP-treated group (*p* < 0.01), indicating a potential synergistic effect between colistin and selenium nanoparticles in suppressing *mexY* expression. The difference between colistin and SeNPs groups was not statistically significant, but Col-SeNP treatment was significantly more effective than both.

In Figure 17c, no significant differences (NS) in *mexY* expression were observed across all treatment groups, including colistin, SeNPs, and Col-SeNPs, when compared to the untreated control. This suggests that under the specific experimental conditions used for this particular dataset, neither colistin nor SeNPs had a notable impact on *mexY* expression. Overall, these findings suggest that selenium nanoparticles, particularly when functionalized with colistin, may downregulate *mexY* expression in certain multidrug-resistant isolates. This effect, observed significantly for isolate B (Figure 17b), was not consistent across all strains tested, indicating that the response may depend on the specific genetic background and resistance mechanisms of each isolate. Full triplicate data and test statistics are provided in the Supplementary Material S1.

## 4. Discussion

The development of synthesized SeNPs is a valuable strategy for counteracting MDR bacterial infections, antifungal-resistant *Candida* spp., and chemoresistance-related cancer cells [52,53]. By way of the colistin-mediated bactericidal and membrane-permeabilizing action and redox-active and cytotoxic activity of synthesized SeNPs, the latter shows enhanced antimicrobial, antifungal, and anticancer activity. This work evaluates their biological activity, that is, their ability to inhibit MDR *P. aeruginosa*, *mexY* gene expression inhibition, enhance antifungal activity, and induce apoptosis in MCF-7 breast cancer cells. The effect of SeNP physicochemical properties, as determined using structural and spectroscopic methods, on their importance in biological interactions and drug applications is also studied. Lastly, the clinical utility of Col-SeNPs, their advantages over conventional treatments, and the agenda for continued research into their translation to the clinic are discussed.

The structural and physicochemical characterization of synthesized SeNPs is highly essential in order to establish their bio-stability, cell interactions, and therapeutic activity [54,55]. In the present study, various analytical techniques, like UV-Vis spectroscopy, AFM, EDX, XRD, FESEM, TEM, and FTIR, were used to determine the synthesis of SeNPs, as well as Col-SeNP properties. The results confirmed the successful synthesis, high purity, and homogenous morphology, as well as effective colistin functionalization that facilitated enhanced bioactivity. UV-Vis spectroscopy showed a distinct surface plasmon resonance peak, proving the existence of stable synthesized SeNPs with tunable size-dependent optical properties [56]. AFM investigations provided three-dimensional topographical data and provided an average height of 10.161 nm with a narrow range, proving nanoparticle size uniformity [57]. FESEM and TEM imaging also ensured a large spherical shape with minimal aggregation, while colistin coating in Col-SeNPs improved nanoparticle dispersity and prevented cluster formation [42]. The XRD analysis ensured the crystalline nature of synthesized SeNPs, and EDX elemental mapping ensured the presence of selenium as the predominant element without detectable impurities, which ensures high purity [58]. FTIR spectroscopy demonstrated information about the functional groups that are accountable for nanoparticle stability, i.e., hydroxyl (-OH), amine (-NH), and carbonyl (-C=O) groups, which are accountable for nanoparticle biocompatibility and bioactivity [59]. These findings emphasize the extensively reported physicochemical properties of Col-SeNPs, which confirm their potential in biomedical applications, like antimicrobial, antifungal, and anticancer therapies. The increase in MDR *P. aeruginosa* also underscores the need for novel antimicrobial therapy [60]. In the current study, it was found that Col-SeNPs exhibited much greater antibacterial activity than SeNPs alone, which was further evidenced by MICs and bacterial viability assays. Enhanced bactericidal activity is a consequence of synergy between colistin and synthesized SeNPs, as demonstrated in previous studies involving colistin combined with nanoparticles, such as bimetallic silver-copper oxide against pan-drug-resistant *P. aeruginosa* [21] and the synthesis of SeNPs [61], in which the outer membrane of bacteria is destabilized by colistin, facilitating increased permeation and the intracellular internalization of nanoparticles [16]. Likewise, when compared to colistin alone, Fuller et al. (2020) [62] found that gold nanoparticles conjugated with colistin significantly increased its antibacterial efficacy against *E. coli*, resulting in a six-fold reduction in the MIC. Upon entry into the bacterial cell, synthesized SeNPs produce ROS, disrupting oxidative homeostasis and interfering with critical metabolic processes, eventually causing irreversible cellular damage [13,14].

One of the good findings was the ability of Col-SeNPs to inhibit *mexY* gene expression, one of the major mechanisms of colistin resistance in *P. aeruginosa* [63]. Efflux pumps, such as *MexXY–OprM*, function to actively efflux antimicrobial substances, lowering drug levels inside cells and enabling resistance. The inhibitory action against *mexY* implies that Col-SeNPs are interfering with the resistance mechanism, thereby reconstituting the sensitivity of bacteria towards colistin. These findings highly indicate the therapeutic potential of Col-SeNPs as an adjuvant therapy for improving colistin efficacy against colistin-resistant *P. aeruginosa* infections. Similar synergistic effects have also been proposed in other recent studies, further confirming the applicability of this strategy [61].

**Figure 18:**
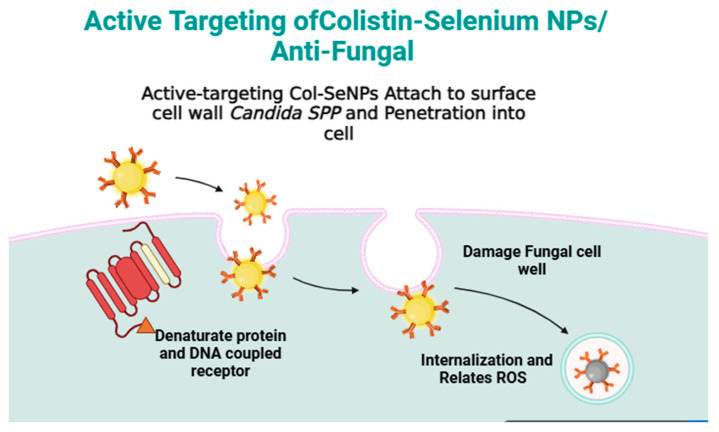
Proposed mechanisms for the antifungal activity induced by Col-SeNPs.

Blocking efflux pumps, like *MexXY–OprM*, is a central achievement in overcoming antimicrobial resistance [64,65]. Since efflux pumps contribute to resistance not only against colistin but also other last-line antibiotics, the potential of Col-synthesis of SeNPs extends beyond *P. aeruginosa*. Similar mechanisms exist in other clinically important Gram-negative pathogens, such as *Acinetobacter baumannii* [66,67,68] and *Klebsiella pneumoniae* [69], for which efflux pump inhibitors have been proposed as viable therapeutic adjuncts. This study thus opens the door for further research into nanoparticle-based treatments to inhibit efflux-mediated resistance in different MDR pathogens. The antifungal activity of Col-SeNPs was tested against azole-resistant *C. krusei*, *C. glabrata*, and *C. albicans* and found to result in the concentration-dependent inhibition of the growth of the fungi. A set of mechanisms is known to exert the antimicrobial effect of the NPs. A mechanistic pathway is proposed (Figure 18).

Col-SeNPs were observed to have much greater antifungal activity than SeNPs alone because of chitosan functionalization. Chitosan enhances nanoparticle interactions with the fungal cell membrane by increasing permeability, as well as intracellular uptake [43]. Selenium has also been reported to interfere with mitochondrial function, destabilize membrane integrity, and induce oxidative stress, thus triggering apoptosis-like cell death [70,71]. Statistically significant antifungal activity at higher concentrations (*p* < 0.0001) proves that Col-SeNPs can be used as a promising alternative or adjunctive treatment for drug-resistant *Candida* infections, for which treatment is limited. In addition to antimicrobial activity, Col-SeNPs also exhibited dose-dependent cytotoxicity towards MCF-7 breast cancer cells with greater activity than SeNPs alone.

Apart from the *Candida* spp., the antifungal use of SeNPs and Col-SeNPs might also be found to be pertinent for combating other clinically important fungal pathogens, such as *Aspergillus fumigatus* [72] and *Penicillium italicum* [73], which are also developing augmenting resistance against antifungals. The SeNP-functionalized chitosan approach can be rationalized to concentrate on ergosterol-dependent pathways, the fungal redox stress response, and biofilm-associated resistance [74,75,76]. This means that investigating the broad-spectrum antifungal efficacy of Col-SeNPs could lead to novel treatment prospects against invasive fungal infections, particularly in immunocompromised individuals.

SeNPs show their anticancer activity through selenium-mediated oxidative stress, mitochondrial injury, and apoptosis induction [15,26]. SeNPs were shown to activate tumor suppressor pathways, such as p53, promote the expression of pro-apoptotic proteins (Bax, caspase-3, caspase-9), and suppress anti-apoptotic proteins (Bcl-2), inducing programmed cell death [14,77]. Moreover, colistin functionalization may also promote the cancer cell internalization of nanoparticles, making them more cytotoxic [78]. The significant reduction in the viability MCF-7 cells at higher doses agrees with previous reports that nanoparticles specifically target cancer cells without inducing death in normal cells [79,80], making Col-SeNPs potential candidates for targeted cancer therapy.

This work extends previous work on SeNPs and other nanometals, since it confirms that the functionalization of colistin significantly increases their antifungal, antimicrobial, and anticancer activities [21,61,81,82]. Tailoring nanoparticles to specifically target efflux pumps and associated proteins may improve efficacy and minimize unwanted effects, ultimately enhancing their potential as a therapeutic strategy [83]. In this work, we hereby provide tangible proof that *mexY* expression is downregulated by Col-SeNPs, thereby making bacteria sensitive to colistin. Furthermore, Col-SeNPs show enhanced antifungal efficacy against azole-resistant *Candida* spp., with a specific emphasis on the role of chitosan functionalization in maximizing nanoparticle penetration and fungicidal effects.

The anticancer use of SeNPs and Col-SeNPs demands their further investigation as a delivery vehicle for targeted delivery and interactions with the tumor microenvironment [84]. The optimization of functionalization protocols can further improve preferential uptake by cancer cells, e.g., through tumor-targeting ligand conjugation or pH-responsive coatings, to allow site-specific drug release [85]. Immunotherapy or conventional chemotherapy medications in combination with Col-SeNPs would further reduce systemic toxicity and improve the therapeutic efficacy. Col-SeNPs and other nanotechnology-delivered therapies could be a new revolution in targeted oncology, as resistance to traditional chemotherapy keeps growing [86].

In the context of the need to create new therapeutic strategies against antimicrobial resistance and chemoresistance, these findings provide ample support for the continuation of developing Col-SeNPs as a second-generation nanotherapeutic. Further work would be aimed at in vivo pharmacokinetics and toxicity and synergy with traditional antimicrobial and anticancer agents to make them more clinically relevant. With further research and optimization, Col-SeNPs show excellent potential to treat global challenges in infectious disease treatment and oncology.

## 5. Conclusions

This study shows Col-SeNPs as a potent nanotherapeutic agent with high antibacterial, antifungal, and anticancer activity. By combining the membrane-permeabilizing property of colistin with the redox property and cytotoxicity of SeNPs, Col-SeNPs showed enhanced inhibitory activity against MDR *P. aeruginosa*, a reduction in *mexY* gene expression, increased antifungal activity against azole-resistant *Candida* spp., and high cytotoxicity against the MCF-7 breast cancer cells. The careful physicochemical characterization attested to their high purity, uniform morphology, and effective colistin functionalization, guaranteeing their stability and biomedical compatibility. The ability of Col-SeNPs to restore colistin susceptibility of MDR *P. aeruginosa* by suppressing efflux pump activity is an important achievements against antibiotic resistance, whereas their improved fungicidal activity indicates their possibility as an alternative or adjunctive antifungal treatment. Additionally, their oxidative-stress-mediated selective cytotoxicity against MCF-7 cells, mitochondrial injury, and induction of apoptosis further validate their efficacy as a nanomedicine candidate to treat cancer. With the urgent need for novel treatments for resistant infections, as well as challenging-to-treat cancers, the present results strongly warrant the further development of Col-SeNPs usingin vivo models, pharmacokinetics, and combined studies with existing therapeutics. Through subsequent research, Col-SeNPs are an extremely promising next-generation nanotherapeutic for addressing very important challenges in cancer treatment and in antimicrobial and fungal infection resistances.

## Figures and Tables

**Table 1:** Primers used in this research.

Primer		Sequence 5′-3′	Annealing Temp. (°C)	Product Size (bp)	Reference
*mexY*	F	5′-CCGCTACAACGGCTATCCCT-3′	60	246	[50]
	R	5′-AGCGGGATCGACCAGCTTTC-3′			
*fbp*	F	5′-CCTACCTGTTGGTCTTCGACCCG-3′	58	284	[51]
	R	5′-GCTGATGTTGTCGTGGGTGAGG-3′			

## Data Availability

The data supporting the findings of this study are available from the corresponding author, Mais E. Ahmed, upon reasonable request.

## Supplementary Materials

The following supporting information can be downloaded at: https://www.mdpi.com/article/10.3390/pharmaceutics17050556/s1, File S1: Microbiology Chart Reports and triplicate data of fold-change of *MexY* gene expression.
