# Supplementary material for: Colistin-Conjugated Selenium Nanoparticles: A Dual-Action Strategy Against Drug-Resistant Infections and Cancer
Source: Pharmaceutics. 2025 Apr 24;17(5):556. doi: 10.3390/pharmaceutics17050556 (PMC12114847; doi:10.3390/pharmaceutics17050556)
Supplement: Supplementary file 1 [file pharmaceutics-17-00556-s001.zip › pharmaceutics-3580730-supplementary.pdf]

Patient Name: R3, . Patient ID: ewgqwgv
Location: Physician:
Lab ID: 1 Isolate Number: 1

Organism Quantity:
Selected Organism : *Candida albicans*

Source: **Collected:**

**Comments:**	

**Identification Information**	**Analysis Time:**	17.77 hours	**Status:**	Final
**Selected Organism**	**Bionumber:**	**Saccharomyces cerevisiae** 4012545025304100		
**ID Analysis Messages**				

Biochemical Details

3	LysA	-	4	lMLTa	-	5	LeuA	+	7	ARG	-	10	ERYa	-	12	GLYLa	-
13	TyrA	+	14	BNAG	-	15	ARBa	-	18	AMYa	-	19	dGALa	+	20	GENa	-
21	dGLUa	+	23	LACa	-	24	MAdGa	+	26	dCELa	-	27	GGT	-	28	dMALa	+
29	dRAFa	+	30	NAGA1	-	32	dMNEa	+	33	dMELa	-	34	dMLZa	(-)	38	lSBEa	-
39	lRHAa	-	40	XLTa	+	42	dSORa	-	44	SACa	+	45	URE	-	46	AGLU	+
47	dTURa	+	48	dTREa	+	49	NO3a	-	51	lARAa	-	52	dGATa	-	53	ESC	-
54	lGLTa	-	55	dXYLa	-	56	LATa	+	58	ACEa	+	59	CITa	-	60	GRTas	-
61	lPROa	-	62	2KGa	-	63	NAGa	-	64	dGNTa	-						

Patient Name: R4, . Patient ID: ERGBDF
Location: Physician:
Lab ID: 2 Isolate Number: 1

Organism Quantity:
Selected Organism : *Candida guilliermondii*

Source: **Collected:**

**Comments:**	

**Identification Information**	**Analysis Time:**	17.77 hours	**Status:**	Final
**Selected Organism**	94% Probability	**Candida sphaerica**		
	**Bionumber:**	4002545225304100		
**ID Analysis Messages**				

Biochemical Details

3	LysA	-	4	lMLTa	-	5	LeuA	+	7	ARG	-	10	ERYa	-	12	GLYLa	-
13	TyrA	-	14	BNAG	-	15	ARBa	-	18	AMYa	-	19	dGALa	+	20	GENa	-
21	dGLUa	+	23	LACa	-	24	MAdGa	+	26	dCELa	-	27	GGT	-	28	dMALa	+
29	dRAFa	+	30	NAGA1	-	32	dMNEa	+	33	dMELa	-	34	dMLZa	+	38	lSBEa	-
39	lRHAa	-	40	XLTa	+	42	dSORa	-	44	SACa	+	45	URE	-	46	AGLU	+
47	dTURa	+	48	dTREa	+	49	NO3a	-	51	lARAa	-	52	dGATa	-	53	ESC	-
54	lGLTa	-	55	dXYLa	-	56	LATa	+	58	ACEa	+	59	CITa	-	60	GRTas	-
61	lPROa	-	62	2KGa	-	63	NAGa	-	64	dGNTa	-						

Patient Name: R7, . Patient ID: WEGWE
Location: Physician:
Lab ID: 3 Isolate Number: 1

Organism Quantity:
Selected Organism : *Candida ciferrii*

Source: **Collected:**

**Comments:**	

**Identification Information**	**Analysis Time:**	17.77 hours	**Status:**	Final
**Selected Organism**	90% Probability	**Cryptococcus albidus**		
	**Bionumber:**	6016145637512130		
**ID Analysis Messages**				

Biochemical Details

3	LysA	-	4	lMLTa	+	5	LeuA	+	7	ARG	-	10	ERYa	-	12	GLYLa	-
13	TyrA	+	14	BNAG	-	15	ARBa	-	18	AMYa	-	19	dGALa	+	20	GENa	(+)
21	dGLUa	+	23	LACa	-	24	MAdGa	-	26	dCELa	-	27	GGT	-	28	dMALa	+
29	dRAFa	+	30	NAGA1	-	32	dMNEa	+	33	dMELa	-	34	dMLZa	+	38	lSBEa	+
39	lRHAa	+	40	XLTa	+	42	dSORa	-	44	SACa	+	45	URE	+	46	AGLU	+
47	dTURa	+	48	dTREa	(-)	49	NO3a	+	51	lARAa	+	52	dGATa	-	53	ESC	-
54	lGLTa	-	55	dXYLa	+	56	LATa	-	58	ACEa	+	59	CITa	-	60	GRTas	-
61	lPROa	+	62	2KGa	+	63	NAGa	-	64	dGNTa	-						

Patient Name:, .
Location: طالبة بحث
Lab ID: 34390

Patient ID: 34390
Physician:
Isolate Number: 1

Organism Quantity:
Selected Organism : Pseudomonas aeruginosa

Source: **Collected:**

**Comments:**	

**Identification Information**	**Analysis Time:** 4.87 hours	**Status:** Final
**Selected Organism**	99% Probability **Pseudomonas aeruginosa**<br>**Bionumber:** 0003053103500252	
**ID Analysis Messages**		

Susceptibility Information	**Analysis Time:** 12.68 hours	**Status:** Final

Antimicrobial	MIC	Interpretation	Antimicrobial	MIC	Interpretation
Ticarcillin	64*	*R	Amikacin	<= 2	S
Ticarcillin/Clavulanic Acid	64	S	Gentamicin	<= 1	S
Piperacillin	64	*R	Tobramycin	<= 1	S
Piperacillin/Tazobactam	16	S	Ciprofloxacin	<= 0.25	S
Ceftazidime	4	S	Pefloxacin		
Cefepime	4	S	Minocycline		
Aztreonam			Colistin	1	S
Imipenem	0.5	S	Rifampicin		
Meropenem	0.5	S	Trimethoprim/ Sulfamethoxazole		

***= AES modified **= User modified**

AES Findings	
**Confidence:**	Consistent with correction

Patient Name:, .
Location: طالبة بحث
Lab ID: 34389

Patient ID: 34389
Physician:
Isolate Number: 1

Organism Quantity:
Selected Organism : Pseudomonas aeruginosa

Source: **Collected:**

**Comments:**	

**Identification Information**	**Analysis Time:** 4.85 hours	**Status:** Final
**Selected Organism**	97% Probability **Pseudomonas aeruginosa**<br>**Bionumber:** 0043453143500252	
**ID Analysis Messages**		

Susceptibility Information	**Analysis Time:** 13.65 hours	**Status:** Final

Antimicrobial	MIC	Interpretation	Antimicrobial	MIC	Interpretation
Ticarcillin	64*	*R	Amikacin	<= 2	S
Ticarcillin/Clavulanic Acid	64	S	Gentamicin	<= 1	S
Piperacillin	64	*R	Tobramycin	<= 1	S
Piperacillin/Tazobactam	16	S	Ciprofloxacin	<= 0.25	S
Ceftazidime	4	S	Pefloxacin		
Cefepime	4	S	Minocycline		
Aztreonam			Colistin	2	S
Imipenem	2	S	Rifampicin		
Meropenem	<= 0.25	S	Trimethoprim/ Sulfamethoxazole		

***= AES modified **= User modified**

**AES Findings**	
**Confidence:**	Consistent with correction

Organism Quantity:
Selected Organism : Pseudomonas aeruginosa

Source: **Collected:**

**Comments:**	

**Identification Information**	**Analysis Time:**	5.82 hours	**Status:**	Final
**Selected Organism**	95% Probability	**Pseudomonas aeruginosa**		
	**Bionumber:**	0043453103500372		
**ID Analysis Messages**				

Susceptibility Information	**Analysis Time:**	12.90 hours	**Status:**	Final

Antimicrobial	MIC	Interpretation	Antimicrobial	MIC	Interpretation
Ticarcillin	64*	*R	Amikacin	<= 2	S
Ticarcillin/Clavulanic Acid	64	S	Gentamicin	2	S
Piperacillin	64	*R	Tobramycin	<= 1	S
Piperacillin/Tazobactam	8	S	Ciprofloxacin	<= 0.25	S
Ceftazidime	8	S	Pefloxacin		
Cefepime	4	S	Minocycline		
Aztreonam			Colistin	1	S
Imipenem	2	S	Rifampicin		
Meropenem	0.5	S	Trimethoprim/ Sulfamethoxazole		

***= AES modified **= User modified**

AES Findings	
**Confidence:**	Consistent with correction

Supplementary data S1

triplicate data of *fold-change of MexY gene expression*.

A

**Treatment**	**Mean ± SD Fold of Change**	**Sig.**	***p* Value**
**Untreated**	1.0 ± 0.0a	**	<0.0001
**Colistin**	0.558 ± 0.07b		
**Se NPs**	0.234 ± 0.043c		
**Col-Se NPs**	0.2 ± 0.01c		

Tukey's multiple comparisons test	Below threshold?	Summary	Adjusted P Value
**Untreated** vs. **Colistin**	Yes	**	<0.0001
**Untreated** vs. **Se NPs**	Yes	**	<0.0001
**Untreated** vs. **Col-Se NPs**	Yes	**	<0.0001
**Colistin** vs. **Se NPs**	Yes	**	<0.0001
**Colistin** vs. **Col-Se NPs**	Yes	**	<0.0001
**Se NPs** vs. **Col-Se NPs**	No	ns	0.7505

B

**Treatment**	**Mean ± SD Fold of Change**	**Sig.**	***p* Value**
**Untreated**	1.0 ± 0.0a	**	<0.0001
**Colistin**	0.34 ± 0.08b		
**Se NPs**	0.37 ± 0.05b		
**Col-Se NPs**	0.12 ± 0.01c		

Tukey's multiple comparisons test	Below threshold?	Summary	Adjusted P Value
**Untreated** vs. **Colistin**	Yes	**	<0.0001
**Untreated** vs. **Se NPs**	Yes	**	<0.0001
**Untreated** vs. **Col-Se NPs**	Yes	**	<0.0001
**Colistin** vs. **Se NPs**	No	ns	0.8638
**Colistin** vs. **Col-Se NPs**	Yes	**	0.0021
**Se NPs** vs. **Col-Se NPs**	Yes	**	0.0009

C

Treatment	Mean ± SD Fold of Change	Sig.	*p* Value
**Untreated**	1.0 ± 0.0a	NS	0.0632
**Colistin**	0.742 ± 0.15a		
**Se NPs**	0.895 ± 0.09a		
**Col-Se NPs**	0.811 ± 0.1a		

Tukey's multiple comparisons test	Below threshold?	Summary	Adjusted P Value
**Untreated** vs. **Colistin**	No	ns	0.0549
**Untreated** vs. **Se NPs**	No	ns	0.6008
**Untreated** vs. **Col-Se NPs**	No	ns	0.1777
**Colistin** vs. **Se NPs**	No	ns	0.3155
**Colistin** vs. **Col-Se NPs**	No	ns	0.8349
**Se NPs** vs. **Col-Se NPs**	No	ns	0.7425
